# Hair cortisol levels are associated with overweight and obesity in the ELSA-Brasil cohort

**DOI:** 10.3389/fendo.2024.1361715

**Published:** 2024-04-09

**Authors:** Karine Lino Rodrigues, Patrícia de Oliveira da Silva Scaranni, Evelyn Nunes Goulart da Silva Pereira, Vivian Vieira Dias da Silva, Raquel Rangel Silvares, Beatriz Peres de Araujo, Cristina Castilho, Maria Inês Schmidt, Maria de Jesus Mendes da Fonseca, Rosane Harter Griep, Anissa Daliry

**Affiliations:** ^1^ Laboratory of Clinical and Experimental Physiopathology, Oswaldo Cruz Foundation, Rio de Janeiro, Brazil; ^2^ Gaffrée and Guinle University Hospital, Federal University of the State of Rio de Janeiro, Rio de Janeiro, Brazil; ^3^ Department of Public Health, Federal University of Rio Grande do Sul, Porto Alegre, Brazil; ^4^ Faculty of Medicine, Federal University of Rio Grande do Sul, Porto Alegre, Brazil; ^5^ National School of Public Health, Oswaldo Cruz Foundation, Rio de Janeiro, Brazil; ^6^ Laboratory of Environmental and Health Education, Oswaldo Cruz Foundation, Rio de Janeiro, RJ, Brazil

**Keywords:** chronic stress, hair cortisol levels, obesity, overweight, ELSA-Brasil

## Abstract

**Introduction:**

Hair cortisol level has recently been identified as a promising marker for detecting long-term cortisol levels and a marker of hypothalamic-pituitary-adrenal cortex (HPA) axis activity. However, research on the association between obesity and an altered cortisol metabolism remains controversial.

**Objective:**

This study aimed to investigate the relationship between hair cortisol levels and overweight and obesity in participants from the Brazilian Longitudinal Study of Adult Health (ELSA-Brasil)

**Methods:**

This was a cross-sectional study involving 2,499 participants from the second follow-up (visit 3, 2017-2019) attending research centers in Rio de Janeiro and Rio Grande do Sul states. Hair samples were collected, and cortisol levels were analyzed using enzyme-linked immunosorbent assay (ELISA) kits. Cortisol levels were classified as low (< 40 pg/mg), medium (40–128 pg/mg), or high (> 128 pg/mg). The participants were classified as eutrophic, overweight, or obese according to their weight (kg) and height (m^2^). Odds ratios (ORs) with 95% confidence intervals (95%CI) were estimated.

**Results:**

Of the 2499 individuals, 30% had eutrophic weight, 40% were overweight, and 30% were obese. Notably, cortisol levels gradually increased with increasing body weight. Among participants with high hair cortisol levels, 41.2% were classified as overweight and 34.2% as obese. Multinomial logistic regression analysis indicated that participants with high cortisol levels were 43% (OR =1.43; 95%CI: 1.02–2.03) more likely to be overweight and 72% (OR =1.72; 95%CI:1.20–2.47) more likely to be obese than participants with low hair cortisol levels. After adjustment for all covariates, high cortisol levels remained associated with obesity (OR = 1.54; 95%CI:1.02–2.31) and overweight (OR =1.33; 95%CI:0.91–1.94).

**Conclusion:**

In the ELSA-Brazil cohort, hair stress were positively associated with overweight and obesity. These results underscore the importance of considering stress and cortisol as potential factors in obesity prevention and intervention efforts, and highlight a novel aspect of the complex relationship between stress and obesity in the Brazilian population.

## 1 Introduction

Obesity and overweight are major public health problems worldwide owing to their profound impact on morbidity and mortality (1). They are the primary risk factors for multiple chronic diseases affecting approximately 1.9 billion overweight and 609 million obese adults worldwide (2). In recent years, a growing body of evidence has underscored the pivotal role of stress in the development and maintenance of obesity (3–6). However, the pathophysiological and molecular mechanisms underlying the interactions between stress and obesity remain poorly understood.

Cortisol is the primary hormone involved in biological responses to chronic stress. Cortisol is a member of the glucocorticoid family and a marker of hypothalamic-pituitary-adrenocortical axis (HPA) activity. Cortisol is known to have the following effects: (i) redistribution of adipose tissue to the abdominal region, (ii) increase of appetite and (iii) increased preference for more palatable foods (7, 8). Higher cortisol levels have been detected in obese patients, and weight and cortisol levels are much higher in individuals who gain weight due to stress (9). In addition, Herhaus et al. (2020) recently showed that cortisol reactivity under stress strongly predicts stress-related eating behavior, which in turn influences body mass index (BMI) (10). Although studies have indicated a correlation between cortisol levels and obesity, not all obese patients have elevated cortisol levels (11). Hohman et al. (2023) showed that obese women had a higher perception of stress, but lower urinary cortisol levels compared to women with normal BMI (12). Furthermore, Kjölhede et al. (2014) showed that overweight and obese children between the ages of 6 and 12 years had lower cortisol levels than normal-weight children (13). Similar results have been observed in individuals with a family history of diabetes, wherein cortisol levels were higher in normal-weight individuals than in overweight/obese individuals (14). Therefore, the results of studies on the association between obesity and altered cortisol metabolism remain controversial.

Hair analysis of cortisol concentration has proven to be a valuable tool for the investigation of mental disorders and stress-related diseases (15), as it allows long-term analysis of cortisol levels and overcomes the limitations of analyzing serum, salivary, or urinary cortisol levels (16). A systematic review examined the correlation between hair cortisol concentration and obesity in children and adolescents and found inconclusive results (17). In contrast, Wester et al. (2014) found higher hair cortisol levels in obese patients than in overweight and normal-weight individuals, whereas no significant difference in hair cortisol levels was found between normal-weight and overweight individuals (18). Due to the limited and inconclusive results regarding long-term cortisol levels as a marker of the stress system that influences body weight, this study aimed to determine whether hair cortisol levels are associated with overweight and obesity in participants of the Longitudinal Study of Adult Health (ELSA-Brasil).

## 2 Materials and methods

### 2.1 Study population and design

This cross-sectional study used data from the Brazilian Longitudinal Study of Adult Health (ELSA-Brasil), a multicenter cohort study that included baseline 15,105 employees from six public higher education and research institutions in the Northeast, South, and Southeast regions of Brazil. Clinical and anthropometric examination data were collected on-site using questionnaires and clinical examinations between August 2017 and December 2019 (19–21).

**Figure 1:**
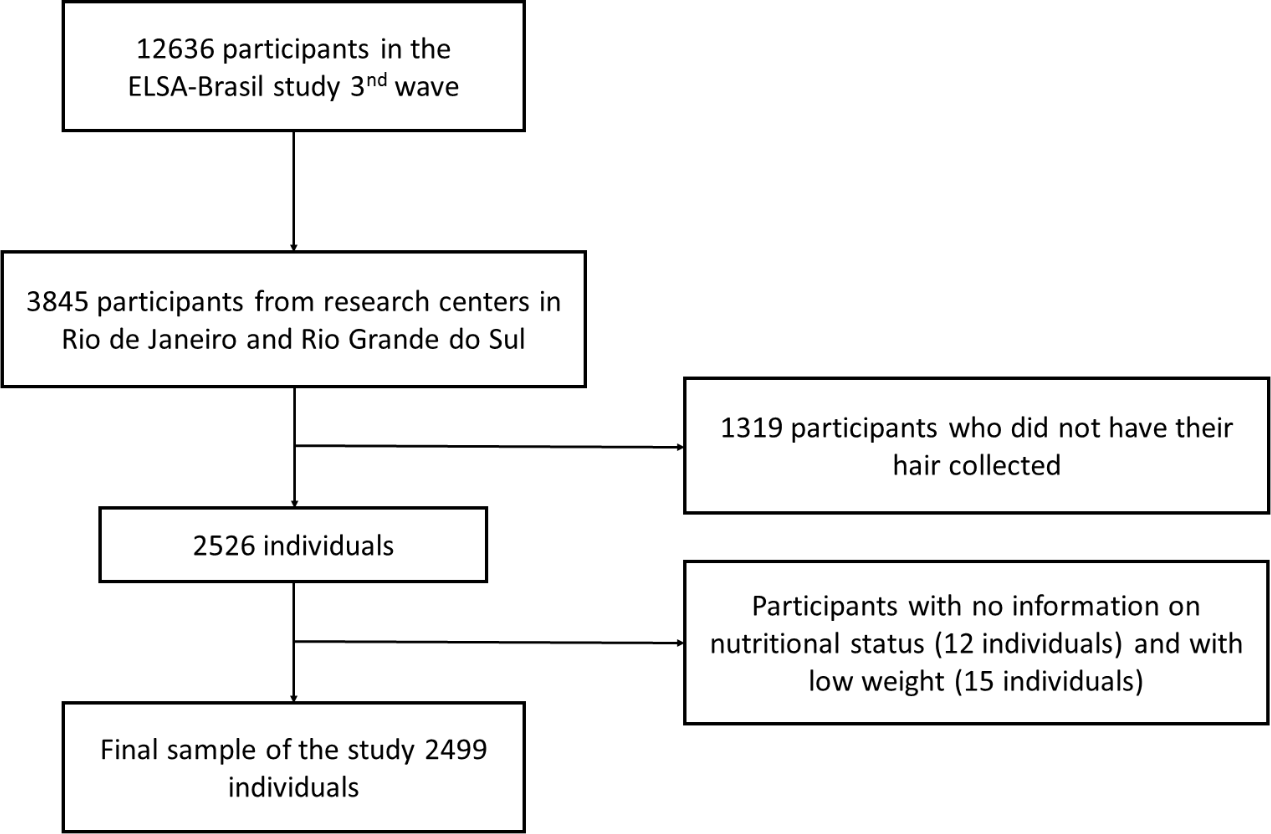
Flowchart detailing the study population selection of participants of ELSA-Brasil study (2017–2019).

For the current study, we used data obtained during the second follow-up of interviews and examinations (Wave 3), that took place between 2017 and 2019 (n=12636). Furthermore, only those from research centers in Rio de Janeiro and Rio Grande do Sul (n=3845) took part in our study. Participants who did not have hair samples collected (n=1319), those with missing data on nutritional status (n=12), or those who were underweight (n=15) were excluded from the analyses. The final sample consisted of 2499 participants (**Figure 1**).

This study was approved by the National Ethics Committee (CONEP) and the local ethics committee. All the participants signed an informed consent form before participating in the ELSA-Brasil study.

#### 2.1.1 Exposure: measuring hair cortisol levels

Hair samples used for the study were cut to a length of approximately 3 cm, which corresponds to 3 months of exposure to cortisol. Hair samples were obtained with scissors from the posterior vertex as close to the scalp as possible. The samples were prepared by washing them twice with isopropanol (2.5 mL) and homogenizing them via repeated inversion for 3 min during each wash. After washing, the samples were stored at room temperature for 2–3 days to allow the complete evaporation of isopropanol. Then, the samples were perforated with scissors (+/- 1 mm) and weighed (10–20 mg) (15, 22, 23).

For cortisol extraction, 1 mL of methanol was added to the tubes containing hair, and the tubes were shaken at 50 °C for 24 h. After the extraction was complete, the supernatant was transferred to a new tube and evaporated at 4 °C until completely dry. The samples were then resuspended in 200 µL of phosphate-buffered saline (PBS) (15, 22, 23).

Cortisol levels in hair samples were analyzed using enzyme-linked immunosorbent assay (ELISA) kit (RE52611-IBL) following the manufacturer’s guidelines. The study population was categorized into three groups: individuals with low (<40 pg/mg), normal (40–128 pg/mg), or high (>128 pg/mg) cortisol levels (24).

#### 2.1.2 Outcome: BMI

Weight (kg) and height (m²) were measured according to standardized protocols (25). Eutrophic participants were defined as BMI < 25 kg/m^2^, overweight as 25 kg/m^2^ ≥ BMI < 30 kg/m^2^, and obese as BMI ≥ 30 kg/m^2^. as defined by the World Health Organization (WHO). BMI was calculated using the following formula: weight (kg) ÷ (height × height) (m).

### 2.2 Covariates

Socio-demographic and socio-economic data included: age group (< 54 years, 55 to 64 years, 65 years and older), race (black, brown, white — we chose to exclude Asians and indigenous as they are not represented in our cohort), alcohol consumption (non-drinker, moderate drinker, excessive drinker — defined as >140 g for women and >210 g for men), smoking habits (non-smoker, ex-smoker, or smoker), physical activity (weak: does not practice physical activity, moderate: <150 min per week of moderate physical activity or walking, high: >150 min per week of moderate physical activity or walking) (24), marital status (with partner, without partner), education level (below high school, completed high school and college, higher), and monthly per capita family income in U.S. dollars (USD, conversion rate: one Brazilian reals ¼ USD) (continuous and weak: does not practice physical activity, medium, and high based on tertiles).

### 2.3 Statistical analyses

Participant characteristics are described as median (interquartile range) for continuous variables and frequency (percentage) for categorical variables. Given the ordinal nature of the stratification groups, we performed trend analysis using the Jonckheere-Terpstra test for continuous variables and the Cox-Mantel-Haenszel test for categorical variables. Multinomial logistic regression analysis was used to examine the relationship between hair cortisol levels (low, medium, or high) and nutritional status (eutrophic, overweight, or obese). All covariates associated with the outcome in the descriptive analyses (p < 0.20) were tested in the adjusted models, and the covariate that contributed the least (largest p value) was removed from the full model. This process was repeated until all remaining covariates were significant (p < 0.05). Variance statistics and the Akaike information criterion (AIC) were used to assess the significance of each variable in the model; lower values indicated the best fit. Collinearity among covariates was tested using the generalized variance inflation factor (VIF). We calculated the odds ratios (ORs) with 95% confidence intervals (95%CIs) for four models: crude (model 1), adjusted for model 1 + age group + sex + race + education level + monthly per capita family income (model 2), and adjusted for model 2 + alcohol consumption + smoking habits + physical activity (model 3). The low-cortisol group was used as the reference category. Statistical significance was set at p<0.05 were considered significant. Data analysis was performed using R version 3.6.2 (R Project for Statistical Computing) and run in R Studio version 1.2.5033 (R Foundation for Statistical Computing, Vienna, Austria).

## 3 Results

**Table 1** details the general characteristics of the 2,499 participants according to their nutritional status (eutrophic, overweight, or obese). We found that 30% of participants were eutrophic, 40% were overweight, and 30% were obese. In addition, the majority of these participants were between 55 and 64 years old, female, white, married, had completed college education, had an average per capita family income, were non-smokers, had moderate alcohol consumption, and had low physical activity levels. We also found no significant differences in age between participant categories. Sex, race, marital status, scholarity, family income per capita, smoking, alcohol consumption, and physical activity differed between the groups according to nutritional status (**Table 1**).

**Table 1:** Description of socio-demographic and health behavior of participants from ELSA-Brasil cohort according to the nutritional status.

	Eutrophic	Overweight	Obesity	P value
	742 (29.7)	1010 (40.4)	747 (29.9)	
**Age range - n (%)**				0.113
< 54 years old	271 (31.7)	352 (41.2)	232 (27.1)	
55 a 64 years old	271 (28.8)	364 (38.6)	307 (32.6)	
65 e over	200 (28.5)	294 (41.9)	208 (29.6)	
**Sex - n (%)**				< 0.001
Men	218 (26.8)	390 (48.0)	204 (25.1)	
Women	519 (31.2)	610 (36.7)	533 (32.1)	
**Race - n (%)**				< 0.001
Black	48 (18.0)	98 (36.7)	121 (45.3)	
Brown	98 (23.2)	195 (46.1)	130 (30.7)	
White	570 (32.8)	694 (40,0)	473 (27.2)	
**Marital status - n (%)**				0.018
With partner	444 (29.5)	640 (42.5)	423 (28.1)	
No partner	298 (30.1)	369 (37.3)	322 (32.6)	
**Scholarity - n (%)**				< 0.001
Basic education or less	40 (22.3)	60 (33.5)	79 (44.1)	
High school	150 (23.2)	266 (41.2)	230 (35.6)	
Higher education or more	547 (33.2)	673 (40.8)	428 (26.0)	
**Family income per capita tercile - n (%)**				< 0.001
Low	211 (24.8)	337 (39.6)	304 (35.7)	
Average	243 (29.1)	360 (43.1)	233 (27.9)	
High	287 (35.7)	311 (38.7)	205 (25.5)	
**Smoking- n (%)**				0.002
Never smoked	480 (31.4)	610 (39.9)	439 (28.7)	
Ex-smoker	182 (24.4)	316 (42.4)	247 (33.2)	
Smoker	79 (35.9)	83 (37.7)	58 (26.4)	
**Alcohol consumption - n (%)**				< 0.001
Don’t drink	212 (25.6)	326 (39.3)	291 (35.1)	
Moderate drinker	461 (32.5)	579 (40.8)	380 (26.8)	
Excessive drinker	57 (27.8)	86 (42.0)	62 (30.2)	
**Physical activity levels - n (%)**				< 0.001
Weak	467 (26.9)	689 (39.7)	580 (33.4)	
Moderate	182 (34.9)	219 (42)	121 (23.2)	
Strong	87 (39.0)	95 (42.6)	41 (18.4)	
**Cortisol levels - n (%)**				< 0.001
Low	420 (33.7)	488 (39.2)	338 (27.1)	
Normal	262 (25.9)	422 (41.8)	326 (32.3)	
High	60 (24.7)	100 (41.2)	83 (34.2)	

**Figure 2:**
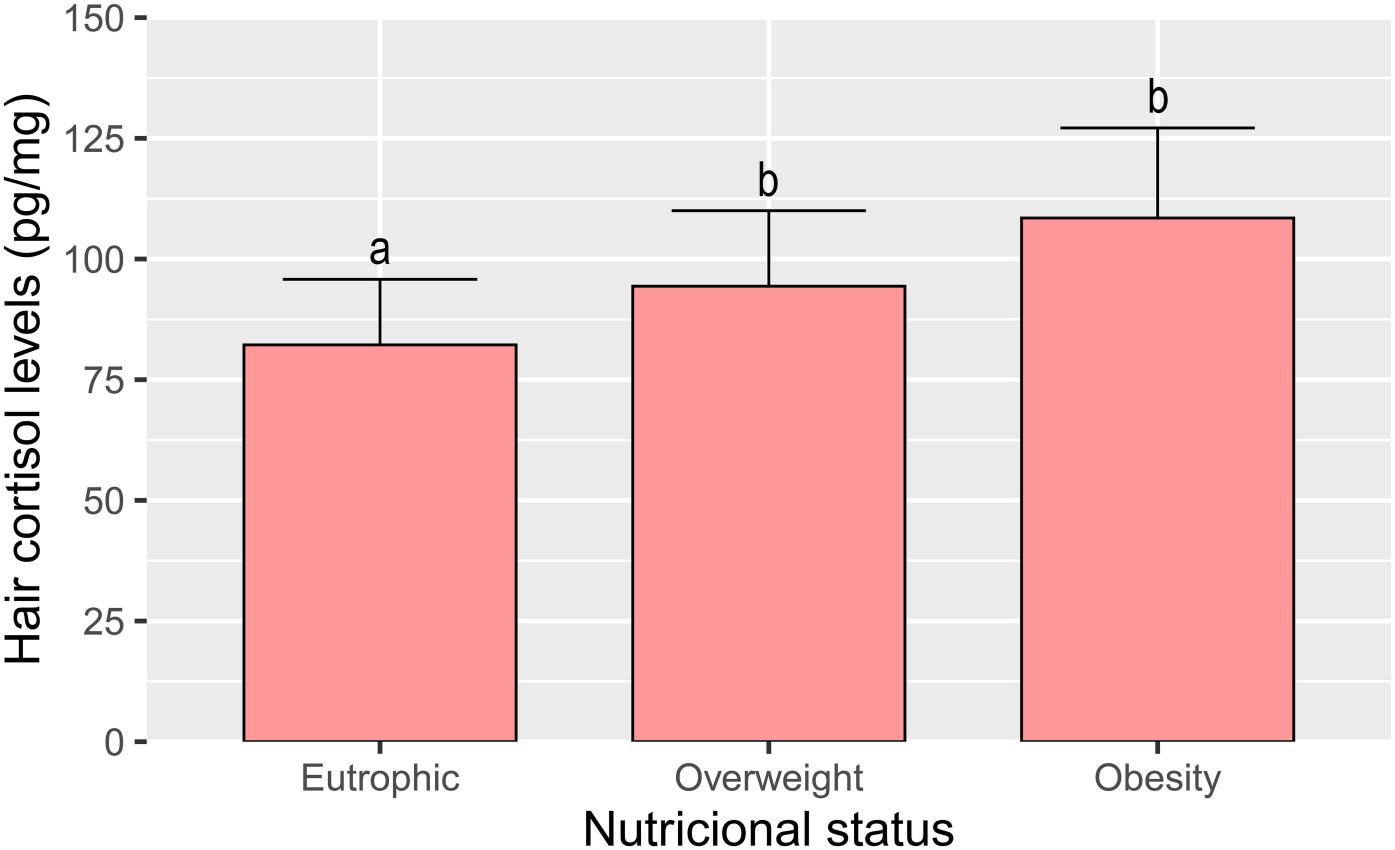
Cortisol levels in hair according to nutritional status (eutrophic, overweight and obesity) of ELSA-Brasil participants (2017–2019). All values are expressed as mean +- SEM. Columns labeled with different letters (a, b) differ significantly (p < 0,001) in the Tukey *post hoc* test.

Regarding cortisol levels, we found that individuals with low hair cortisol levels were 34% eutrophic, 39% overweight and 27% obese, individuals with normal levels were 26% eutrophic, 42% overweight and 32% obese, and individuals with high cortisol levels were 25% eutrophic 41% overweight and 34% obese. When evaluating hair cortisol levels in participants, we also demonstrated an increase in cortisol levels with an increase in body weight; however, hair cortisol levels remained similar in both overweight and obese participants (**Figure 2**).

Additionally, in the multinomial logistic regression analysis, we found that among participants with high hair cortisol levels, 41.2% were classified as overweight and 34.2% as obese (**Table 2**). Participants with high cortisol levels were 43% (OR =1.43; 95%CI: 1.02–2.03) more likely to be overweight and 72% (OR =1.72; 95%CI:1.20–2.47) more likely to be obese than participants with low hair cortisol levels. After adjustment for all covariates, high cortisol levels remained significantly associated with obesity (OR = 1.84; 95%CI:1.24–2.71) and overweight (OR =1.46; 95%CI:1.01–2.11) (**Table 2**).

**Table 2:** Crude and adjusted multinomial logistic regression, with reference to nutritional status and hair cortisol levels in participants of the ELSA-Brasil (2017–2019).

Model 1 (crude)	Low cortisol	Overweight - OR(IC95%)	Obesity - OR(IC95%)
		Reference	Reference
	**Normal cortisol**	**1.39 (1.13–1.70)**	**1.55 (1.24–1.92)**
	**High cortisol**	**1.43 (1.02–2.03)**	**1.72 (1.20–2.47)**
	**AIC**	5426.93	
**Model 2**	**Low cortisol**	Reference	reference
	**Normal cortisol**	**1.34 (1.09–1.65)**	**1.48 (1.18-1.85)**
	**High cortisol**	**1.47 (1.02–2.11)**	**1.77 (1.21-2.59)**
	**AIC**	5113.84	
**Model 3**	**Low cortisol**	Reference	reference
	**Normal cortisol**	**1.34 (1.09–1.66)**	**1.47 (1.17–1.86)**
	**High cortisol**	**1.46 (1.01–2.11)**	**1.84 (1.24–2.71)**
	**AIC**	5032.01	

## 4 Discussion

The relationship between cortisol level and nutritional status and their underlying mechanisms remains controversial. This study used hair samples to measure hair cortisol levels, which provides a more reliable and long-term assessment of cortisol exposure than traditional methods such as serum, saliva, or urine cortisol levels. Hair cortisol analysis is being increasingly optimized and applied in both research and medicine (26–28) as it provides an objective and unique biomarker for analyzing endogenous cortisol levels and shows that the deposition of compounds and their metabolites in hair during growth allows retrospective quantification (29). In the present study, we investigated the association between hair cortisol levels and nutritional status among participants from the ELSA-Brasil cohort. High hair cortisol levels were found in 41.2% of the participants classified as overweight and 34.2% as obese. Multinomial logistic regression analysis showed that participants with higher hair cortisol levels had a 43% increased chance of being overweight and were 72% more likely to be obese than those with low hair cortisol levels. Additionally, in the descriptive analysis, we find differences in sex, race, marital status, scholarity, family income per capita, smoking, alcohol consumption, and physical activity between groups according to nutritional status. The relationship between socio-demographic and health behaviors, such as addiction, and cortisol have been previously shown (30, 31), however, in our study, those variables were not accounting for the association between hair cortisol and BMI, as shown in the multinomial logistic regression analysis.

Similar to our study, Jackson et al. (2017) observed in subset of subjects from the English Longitudinal Study of Ageing (ELSA), including 2,527 men and women, aged ≥50 years, that hair cortisol levels were significantly elevated in participants with obesity (32). Of note, high hair cortisol levels were also positively associated with the persistence of obesity, as assessed by a 4-year follow-up (32). It is worth mentioning that the ELSA cohort predominantly comprised white British participants (98%). On the other hand, our study included a diverse population, from different regions of Brazil, which were 11% black, 18% brown, and 71% white. Thus, our study design increases the generalizability of the findings. Also, our data confirm the findings of Chan et al. (2014), who showed that obese individuals aged 28–67 years have higher hair cortisol levels than healthy individuals, and that this increase correlates with the increase in BMI (33). However, Chan et al. (2014) evaluated only 57 participants, including 39 non-obese and 18 obese individuals, which is a limitation of this study (33).

Although some studies have shown an association between cortisol and obesity, the molecular mechanisms that trigger this association remain unknown. In a systematic review, Rodriguez et al. (2015) demonstrated that greater abdominal fat is associated with greater responsiveness of the HPA axis, which is reflected in morning awakening and acute reactivity to stress. They also showed a marked upregulation of cortisol production when adipocytes were examined; however, it remains to be elucidated how adipocyte cortisol metabolism affects circulating cortisol levels (34). One possible explanation for this effect is that activation of the HPA axis is triggered by the release of corticotropin-releasing hormone and arginine vasopressin from neurons in the paraventricular nucleus of the hypothalamus. Both factors are secreted into the pituitary portal system to act on the corticotrophs of the anterior pituitary. This results in the release of adrenocorticotropin from the pituitary gland, which, in turn, stimulates the adrenal gland to release glucocorticoids. At normal body weight, HPA axis activity is modulated by the closed negative feedback effects of glucocorticoids in the brain and pituitary glands. This negative feedback is impaired in individuals with abdominal or visceral obesity. The attenuated negative feedback effect of cortisol results in elevated cortisol levels and increased cortisol secretion in response to stress (9).

Long-term exposure to psychosocial stressors also activates the sympathetic nervous system, which in turn could participate in the development of obesity and metabolic syndrome. Cortisol not only influences fat deposition, but is also associated with changes in the amount and type of food eaten (35–39). People tend to eat more when they are stressed (40, 41), but some studies have observed reduced food intake during acute stress (39). Studies suggest that the sensitivity of central reward circuitry to foods in which the mesocorticolimbic pathway plays a central role is lower in stressful situations, which may increase the preference for “comfort foods”, i.e. foods that are more palatable and higher in energy (40, 42–45). Although the change in food consumption is an addictive response more related to the stress-obesity interface, it brings us a warning sign for other addictive behaviors related to mesolimbic dopaminergic system and hypercortisolism, such as the consumption of alcohol and drugs in individuals affected by stress (46, 47). It is believed that the dysregulation of the HPA axis through chronic stress is responsible for the ability to increase the body’s vulnerability to these substances (48). Elman et al. (2003) demonstrated that cortisol administration led to increased craving for cocaine in cocaine-dependent individuals compared to those receiving placebo, suggesting that cortisol may be associated with drug dependence (49). Furthermore, Duplessis-Marcotte et al. evaluated during the pandemic (2020-2021) the effect of stress on alcohol consumption in healthy individuals and observed that alcohol use increased only in people with high concentrations of hair cortisol, and that this increase in alcohol consumption remained high one year after (50). The intra-individual differences in physiological responses to cortisol (more food vs. less food; craving for palatable food, induction of other addictions) could therefore explain the different results regarding the relationship between cortisol and obesity found in the literature.

This study has some limitations. First, owing to its cross-sectional design, this study may have limited its ability to demonstrate causality. It can only demonstrate associations, but not causal relationships, between hair cortisol levels and overweight or obesity. Since the exposure (cortisol levels) and the outcomes (nutritional status) were studied in the same patients at the same time, the study design does not allow differentiation between cause and effect. Although several covariates were adjusted for in this study, unmeasured confounders may have influenced the observed associations. Further research, including longitudinal or experimental studies is required to confirm and better understand the association between cortisol levels and obesity.

## 5 Conclusion

In conclusion, we addressed an important and relevant research question related to the association between cortisol levels and obesity, both of which are major public health problems worldwide. This study allowed a thorough examination of the relationship between hair cortisol levels and different nutritional status categories (eutrophic, overweight, and obese). These findings are consistent with previous research suggesting a positive association between cortisol levels and overweight/obesity and add to the existing body of evidence on this topic. In addition to contributing to health disparities, obesity in individuals with elevated cortisol levels is a critical step in the design and implementation of health-promoting interventions.

## Data availability statement

The original contributions presented in the study are included in the article/supplementary materials, further inquiries can be directed to the corresponding author.

## Ethics statement

The studies involving humans were approved by National Ethics Committee (CONEP). The studies were conducted in accordance with the local legislation and institutional requirements. The participants provided their written informed consent to participate in this study.

## Author contributions

KR: Conceptualization, Data curation, Formal analysis, Investigation, Methodology, Resources, Validation, Visualization, Writing – original draft, Software. PS: Data curation, Formal analysis, Software, Writing – original draft. EP: Data curation, Formal analysis, Software, Writing – original draft. Vd: Formal analysis, Writing – original draft. RS: Formal analysis, Writing – original draft. Bd: Formal analysis, Writing – original draft. CC: Formal analysis, Writing – original draft. MS: Formal analysis, Writing – original draft. Md: Formal analysis, Writing – original draft. RG: Conceptualization, Data curation, Formal analysis, Funding acquisition, Investigation, Methodology, Resources, Validation, Visualization, Writing – original draft, Writing – review & editing. AD: Conceptualization, Data curation, Formal analysis, Funding acquisition, Investigation, Methodology, Project administration, Resources, Supervision, Validation, Visualization, Writing – original draft, Writing – review & editing.
